# Giant cell tumor of the capitate: an unusual case with 10 years follow-up

**DOI:** 10.1051/sicotj/2015018

**Published:** 2015-07-10

**Authors:** Serda Duman, Hakan Sofu, Yalkin Camurcu, Sarper Gursu, Ramadan Oke

**Affiliations:** 1 Diyarbakir Selahaddin Eyyubi State Hospital 21100 Diyarbakir Turkey; 2 Erzincan University Faculty of Medicine 24030 Erzincan Turkey; 3 Devrek State Hospital 67800 Zonguldak Turkey; 4 Baltalimani Bone and Joint Diseases Hospital 34470 Istanbul Turkey

**Keywords:** Giant cell tumor, Capitate

## Abstract

Giant cell tumor of the small bones, particularly the carpal bones of the hand, is exceedingly rare. We present a case report of giant cell tumor of the capitate in a 24 year-old female with 10 years postoperative follow-up. Although carpal bones are extremely unusual location, orthopedic surgeons should always keep in mind that differential diagnosis must include giant cell tumor of bone whenever an expansile osteolytic lesion with well-defined but nonsclerotic margins is identified in a young adult with closed physes.

## Introduction

Giant cell tumor of bone (GCTOB) is defined as a benign but locally aggressive primary bone tumor which generally involves the meta-epiphyseal region of long bones, mostly around the knee joint and distal radius of skeletally mature individuals [1]. Clinical presentation and behavior of a GCTOB may vary from a static and symptomless lesion to a very destructive tumor with extensive bone and soft tissue expansion [1]. Giant cell tumor of the small bones, particularly the carpal bones of the hand, is exceedingly rare. A thorough review of the literature revealed very limited numbers of reported primary GCTOB of the capitate [2–4]. We present a case report of GCTOB of the capitate with 10 years follow-up. Our patient was treated with en bloc resection involving the whole capitate, the proximal 1 cm of the third and fourth metacarpals, and the articular surfaces of the trapezium, trapezoid, triquetrum, and hamate. Immediate reconstruction with bicortical autogenous iliac bone graft was also performed during the same surgery. Clinical assessment of the patient at the latest follow-up consisted of range of motion measurements, Mayo wrist score, quick disability of arm shoulder hand questionnaire (Quick-DASH), Gartland and Werley scoring, and plain X-rays and magnetic resonance imaging (MRI) for radiographic evaluation.

## Case

**Figure 1.**
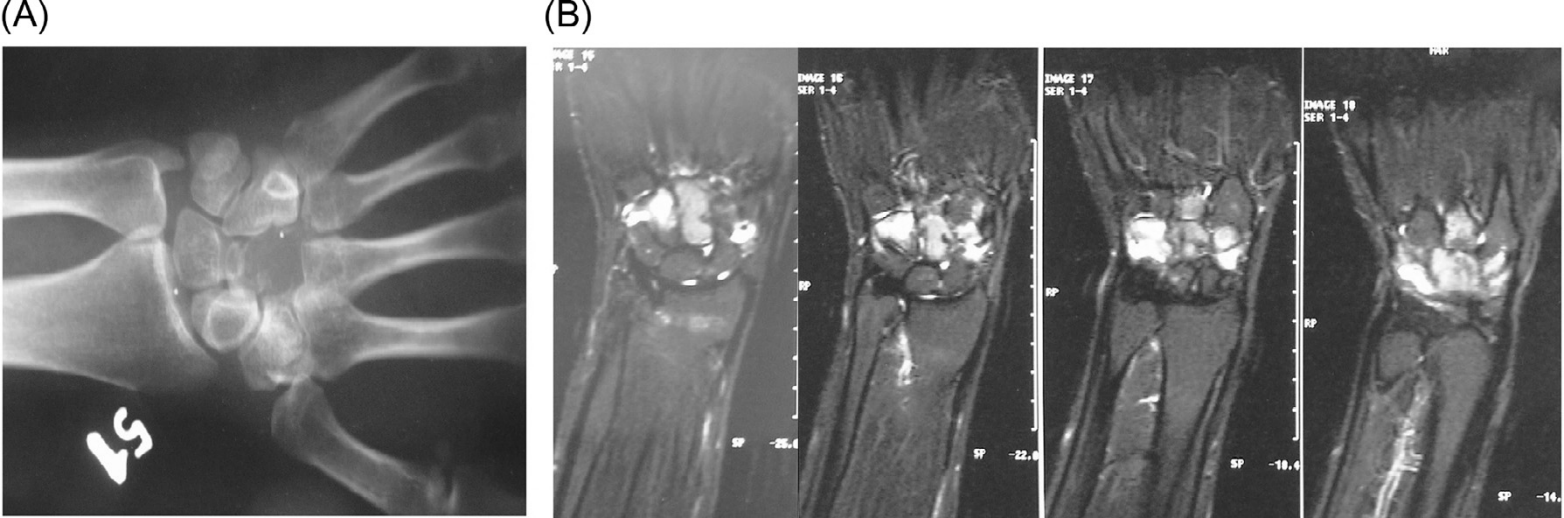
(A) Preoperative roentgenogram of the left wrist, (B) preoperative MRI of the wrist.

**Figure 2.**
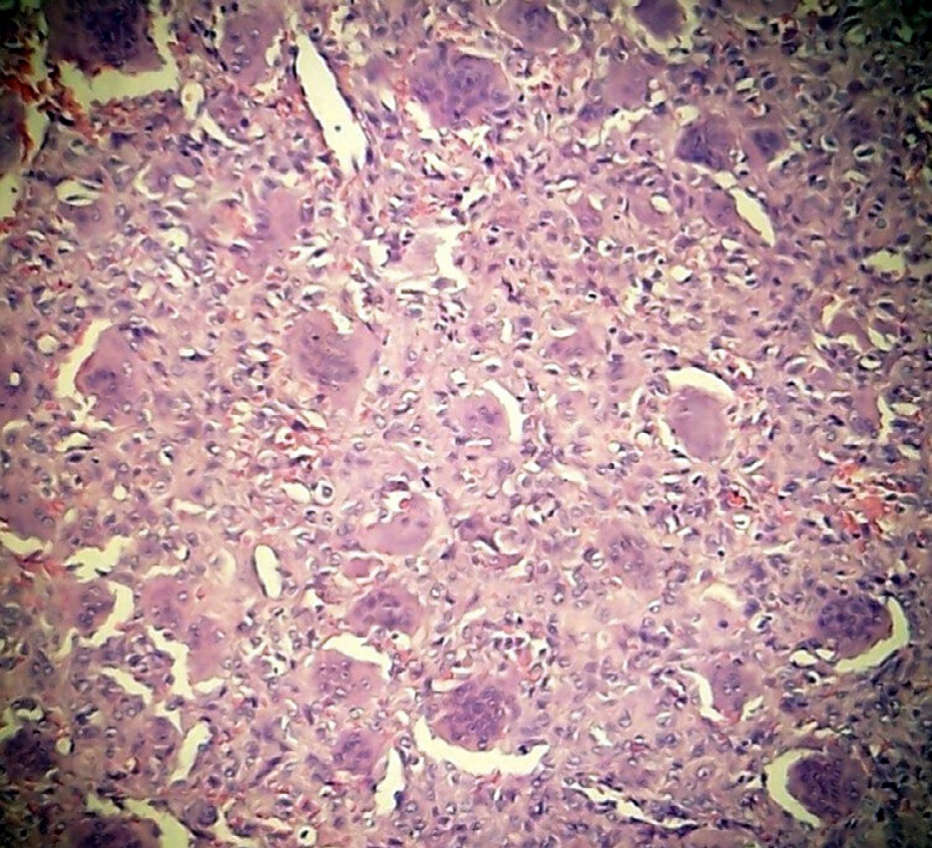
Histological photomicrograph (hematoxylin-eosin stain) of the biopsy specimen.

**Figure 3.**
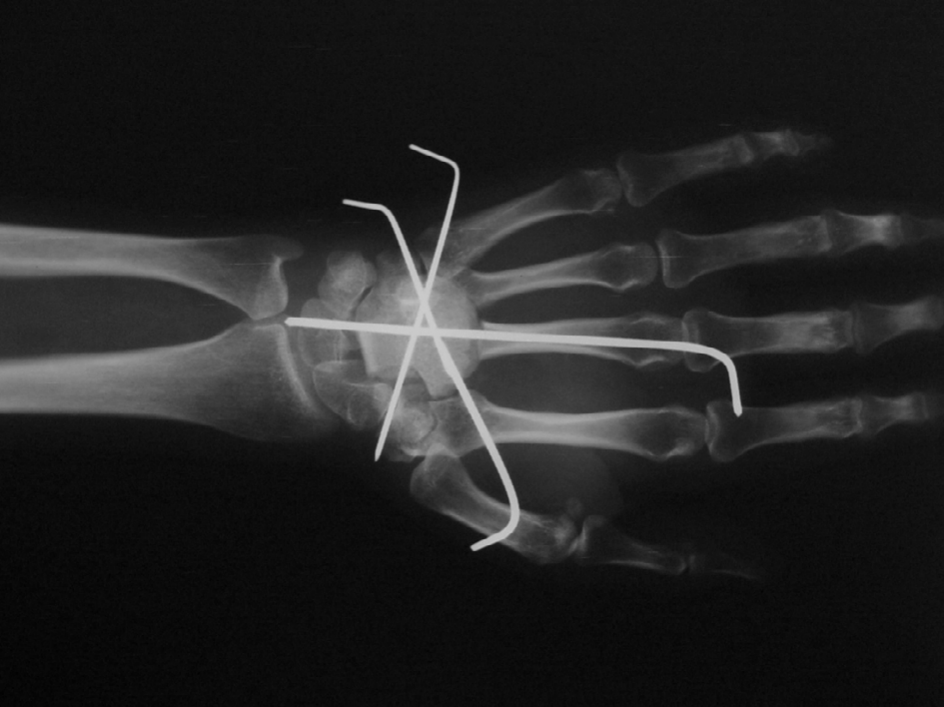
Reconstruction of the resultant defect and K-wire fixation.

A 24-year-old female patient with a 2 × 2 cm wide mild swelling on the dorsum of her left hand, which was painful especially during dorsiflexion and strong grip, was admitted to the orthopedic department. The pain and swelling appeared during her third trimester of pregnancy, persisted for nearly 4 months up to the first admission to the orthopedic department, and she had a 2-month-old baby at the first clinical evaluation. There was no past medical history of trauma or previous treatment. Physical examination revealed the swelling and tenderness on the dorsum of the hand in the area above the carpometacarpal joints. Routine laboratory blood test results were within normal limits. Plain roentgenograms showed a lytic lesion that involved the capitate almost totally except the proximal pole but surrounding bones were normal (Figure 1A). Magnetic resonance imaging revealed a neoplastic lesion expanding beyond the cortices both dorsally and palmarly, eroding the dorsal edge of the third metacarpal bone, and causing edema of hamate and trapezium (Figure 1B). We performed an open biopsy which resulted in the diagnosis of GCTOB (Figure 2). We also conducted a radiologic bone survey and computed tomography (CT) of the chest revealing no pathology, however we postponed a bone scintigraphy because she had a 2-month-old baby and wanted to breastfeed thus avoiding radioactive material. For the definitive treatment, en bloc resection and reconstruction of the resultant defect were performed. The capitate was resected together with proximal 1 cm of the third and fourth metacarpal bones as well as the articular surfaces of the trapezium, trapezoid, triquetrum, and hamate. Scaphoid and lunate were left intact. The resultant defect was reconstructed by using a bicortical iliac bone autograft. Fixation was achieved with K wires (Figure 3). A short arm cast was also applied. Histological examination of the excised specimen confirmed the diagnosis. She had a problem-free healing period. The pins and the cast were removed at the end of 2 months postoperatively.

**Figure 4.**
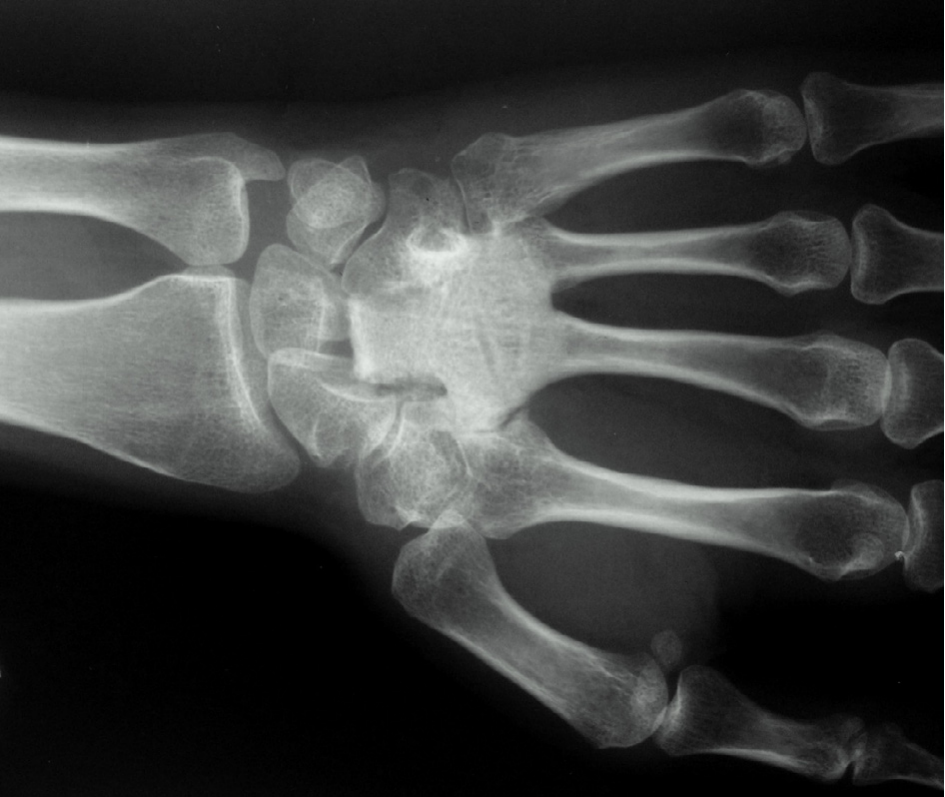
Roentgenogram of the operated wrist at 6-month follow-up.

At the 6-month follow-up clinical visit, she had no pain; flexion and extension of the operated wrist were 30° each, and 20° of radial and 15° of ulnar deviation could be achieved. The graft was incorporated with the third and fourth metacarpal bones, as well as the surrounding carpal bones (Figure 4). The control MRI was obtained and no local recurrence was detected. The previously postponed bone scintigraphy was also performed, and revealed no pathologic findings except the left wrist which was considered to be due to the uncompleted healing process.

**Figure 5.**
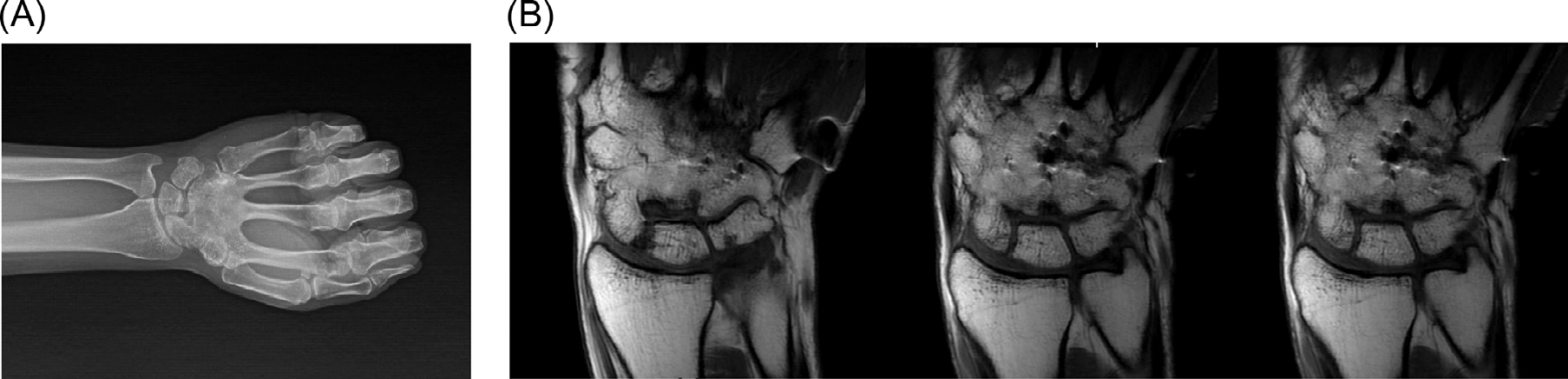
(A) Roentgenogram of the operated wrist at 10-year follow-up, (B) MRI of the operated wrist at 10-year follow-up.

At the 10-year follow-up clinical visit, she had no pain. Quick-DASH score was measured as 27.3 points. Mayo wrist score was 75 points. Gartland and Werley score was 7. The control plain roentgenogram and MRI were obtained, and no local recurrence was detected (Figures 5A and 5B).

## Discussion

Giant cell tumor of bone is still considered by many of the orthopedic surgeons, as a disease that is difficult to treat due to a high incidence of local recurrence and potential to metastasize to lungs or mediastinal nodes. Although it is one of the common primary tumors of the human skeleton and has been discussed widely in the orthopedic literature, controversies regarding the most effective methods of treatment and prevention of its recurrence still continue. The clinical behavior is benign in the majority of the cases; however, metastases in the form of benign pulmonary implants rather than classical metastatic lesions associated with malignant tumors can be diagnosed in 2% of the cases. Giant cell tumor of the small bones, particularly the carpal bones of the hand, is exceedingly rare. We present a case of primary GCTOB of the capitate in a 24-year-old female patient with 10-year postoperative clinical follow-up results.

Intralesional resection and curettage, adjuvants, grafting, filling the defect with bone cement, marginal or wide resection, and even amputation have been discussed as the treatment options in such patients [1, 5]. Adjuvant therapies may include burring, hydrogen peroxide, phenol, alcohol, and polymethylmethacrylate. However, there is still no consensus on the ideal treatment modality. Recurrence rates following intralesional excision with curettage have been reported ranging from 10% to 40% [1, 5]. Campanacci et al. [1] reported that differing rates up to 90% of local recurrences following surgical treatment of GCTOB appeared within the first 3 years after surgery depending on the grade and mode of treatment. The lesion that we resected in our case was Grade 3 according to their classification. A meta-analysis comparing the local control of the tumor with or without adjuvants applied during the surgical resection of the lesion demonstrated no significant difference [5]. Howard and Lassen [2] reported recurrence following resection in their patient with GCTOB of the capitate and they concluded that the recommended treatment of this tumor, if it occurred within the carpus, was resection of the carpus with intercarpal arthrodesis if the distal row was involved or proximal row carpectomy if the scaphoid or lunate was involved. Wilson et al. [4] however successfully treated his patient by performing a combination of intralesional curettage, high-speed burring, cryosurgical ablation, and bone grafting. On the other hand, besides the risk of recurrence, limb function is usually compromised in such patients postoperatively because of the periarticular location of most lesions. In a 28-year-old female patient, McDonald and Schajowicz [3] resected the capitate but no reconstruction was performed. He reported excellent hand function without recurrence at 5-year follow-up. In our case, the tumor had expanded beyond both the dorsal and the palmar cortices as well as the intra-articular space. We performed en bloc resection of the tumor together with intercarpal and partial carpometacarpal arthrodesis. Restriction of movements versus decreasing the risk of recurrence may be discussed. However, sparing the radiocarpal joint permitted a fair range of motion in the operated wrist joint postoperatively. Our patient was fully functional with no complaints during her daily activities after 10 years from surgery.

The multicentric disease incidence is less than 1%, therefore a systemic testing in an asymptomatic individual is not routinely advised. A preoperative chest CT and a scintigraphic bone survey are the clinical tools in case of any requirement for a systemic testing. Our patient did not have any clinical signs of disseminated disease preoperatively. Furthermore, the morbidity to her or to her unborn child would be much higher than the potential benefit of such a procedure. Therefore, we did not perform a bone survey preoperatively. After the birth of her child, our patient was breastfeeding and wanted to avoid radioactive injection until she stopped breastfeeding. We performed a bone scintigraphy at the 6-month follow-up visit which revealed no pathologic findings indicating any other focus.

In conclusion, although carpal bones are extremely unusual location, orthopedic surgeons should always keep in mind that the differential diagnosis leading the physician to the most appropriate treatment must include GCTOB whenever an expansile osteolytic lesion with well-defined but nonsclerotic margins is identified in a young adult with closed physes.

## Conflict of interest

There are not any conflicts of interest regarding preparation, submission and publication of this paper.
